# Trends in intracranial meningioma incidence in the United States, 2004‐2015

**DOI:** 10.1002/cam4.2516

**Published:** 2019-09-01

**Authors:** Dong‐Dong Lin, Jia‐Liang Lin, Xiang‐Yang Deng, Wei Li, Dan‐Dong Li, Bo Yin, Jian Lin, Nu Zhang, Han‐Song Sheng

**Affiliations:** ^1^ Department of Neurosurgery The Second Affiliated Hospital and Yuying Children's Hospital of Wenzhou Medical University Wenzhou China; ^2^ Department of Orthopaedics The Second Affiliated Hospital and Yuying Children's Hospital of Wenzhou Medical University Wenzhou China

**Keywords:** age‐adjusted incidence, demographic and tumor characteristics, meningioma, SEER, trends

## Abstract

**Background:**

Meningioma incidence was reported to have risen substantially in the United States during the first decade of the 21st century. There are few reports about subsequent incidence trends. This study provides updated data to investigate trends in meningioma incidence by demographic and tumor characteristics at diagnosis in the United states from 2004 to 2015.

**Methods:**

Trends in meningioma incidence were analyzed using data from the Surveillance, Epidemiology, and End Results‐18 (SEER‐18) registry database of the National Cancer Institute. The joinpoint program was used to calculate annual percent change (APC) in incidence rates.

**Results:**

The overall incidence of meningioma increased by 4.6% (95% CI, 3.4‐5.9) annually in 2004‐2009, but remained stable from 2009 to 2015 (APC, 0; 95% CI, −0.8 to 0.8). Females (10.66 per 100 000 person‐years) and blacks (9.52 per 100 000 person‐years) had significant predominance in meningioma incidence. Incidence in many subgroups increased significantly up to 2009 and then remained stable until 2015. However, meningioma incidence in young and middle‐aged people increased significantly throughout the entire time period from 2004 to 2015 (APC: 3.6% for <20‐year‐olds; 2.5% for 20‐39‐year‐olds; 1.8% for 40‐59‐year‐olds). The incidence of WHO II meningioma increased during 2011‐2015 (APC = 5.4%), while the incidence of WHO III meningioma decreased during 2004‐2015 (APC = −5.6%).

**Conclusion:**

In this study, the incidence of meningioma was found to be stable in recent years. Possible reasons for this finding include changes in population characteristics, the widespread use of diagnostic techniques, and changes in tumor classification and risk factors in the US population.

## Graphical abstract

We evaluated trends in meningioma incidence according to demographic and tumor characteristics at diagnosis in the United States during 2004‐2015. We found the incidence of meningioma increased significantly in 2004‐2009 and became stable in 2009‐2015. It challenged the prevailing notion that the US meningioma incidence has increased for decades.

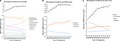

## 1 INTRODUCTION

Meningiomas are generally benign tumors that originate in cerebral dura mater and can grow at any site, especially at the skull vault and the skull base, with 10% of meningioma located in the spinal cord.^1^ Although most meningiomas are asymptomatic^2^ and patients often only experience mild headaches in the early stages, there are a large number of complications and poor functional outcomes when tumors progress. According to the latest Central Brain Tumor Registry of the United States (CBTRUS) statistical report,^3^ meningioma was the most frequently reported histologic type, accounting for 37.1% of all CNS tumors and 53.1% of nonmalignant CNS tumors from 2011 to 2015. Meningioma also has the highest incidence rate, that is, 8.33 per 100 000 person‐years in among CBTRUS histology groupings and an estimated 31 990 new cases in 2019.

Few studies have focused on the trends in the incidence of meningioma. A study of patients aged over 65 years old with meningioma found nonmalignant meningioma incidence increased significantly for both females (APC = 4.69%) and males (APC = 4.79%) from 2005 to 2009, whereas the incidence of malignant meningioma decreased significantly for females (APC = −5.45%) and males (APC = −2.88%) during 2005‐2015.^4^ Furthermore, Kshettry et al revealed that WHO II meningioma incidence increased by 3.6% per year from 2004 to 2010 and WHO III meningioma incidence decreased by 5.4% per year during 2000‐2010.^5^ However, these studies did not cover all populations or all types of meningioma. A report from the UK demonstrated that the meningioma incidence remained stable for the local population over the study period.^6^ However, comprehensive investigations on the trends in meningioma incidence in the United States are lacking.

In our study, we used data from 2004 to 2015 of the SEER registry database to provide an updated report focusing on meningioma incidence trends according to demographic and tumor characteristics at diagnosis.

## 2 METHODS

### 2.1 Data sources

Data on meningioma incidence between 2004 and 2015 were extracted from the SEER‐18 registry database,^7^ which contains cases from 18 high‐quality registries (San Francisco, Connecticut, Detroit, Hawaii, Iowa, New Mexico, Seattle, Utah, Atlanta, San Jose‐Monterey, Los Angeles, Alaska Native Registry, Rural Georgia, California excluding SF/SJM/LA, Kentucky, Louisiana, New Jersey, and Georgia excluding ATL/RG), covering about 27.8% of the US population. Complete records of meningioma have been available since 2004, and we therefore carried out the analysis using data from 2004 to 2015 to ensure that the analysis was complete and up to date. Our study included patients diagnosed with malignant or nonmalignant meningioma according to the International Classification of Diseases for Oncology, Third Edition (ICD‐O‐3) histology codes 9530‐9535 and 9537‐9539.

### 2.2 Patients’ characteristics

We investigated trends in meningioma incidence according to demographic and tumor characteristics obtained from medical records of various registries. Demographic characteristics mainly included sex, race, and age at diagnosis (divided into the following subgroups <20, 20‐39, 40‐59, 60‐79, and ≥80 years old).

Tumor characteristics included histologic types, WHO grade, and tumor size. Histologic types were classified into meningioma NOS, meningothelial, fibrous, psammomatous, angiomatous, hemangioblastic, transitional, clear cell, atypical meningioma, and meningeal sarcomatosis by ICD‐O‐3 codes (Table 1). Only the first matching record and tumors located in cerebral meninges (ICD‐O‐3 topography code C70.0), meninges NOS (ICD‐O‐3 topography code C70.9), or the brain (ICD‐O‐3 topography code C71) were included. Meningiomas were divided into grade I, grade II, and grade III according to National Comprehensive Cancer Network guidelines and ICD‐O‐3 behavior code. Collaborative Staging codes (CS) were used to determine tumor size during 2004‐2015. Diagnostic confirmation was provided by the special code of the SEER database.

**Table 1:** Definitions of meningioma histology groups: The SEER‐18 registry database

Histology group	ICD‐O‐3 code	Specific histology classification
Meningioma, NOS	9530	Meningioma, NOS; Meningiomatosis, NOS; Meningioma, malignant
Meningothelial meningioma	9531	Meningothelial meningioma; Meningothelial meningioma, borderline; Meningothelial meningioma, malignant
Fibrous meningioma	9532	Fibrous meningioma; Fibrous meningioma, malignant
Psammomatous meningioma	9533	Psammomatous meningioma
Angiomatous meningioma	9534	Angiomatous meningioma; Angiomatous meningioma, malignant
Hemangioblastic meningioma	9535	Hemangioblastic meningioma; Hemangioblastic meningioma malignant
Transitional meningioma	9537	Transitional meningioma; Transitional meningioma, malignant
Clear cell meningioma	9538	Clear cell meningioma, benign; Clear cell meningioma; Papillary meningioma
Atypical meningioma	9539	Atypical meningioma, benign; Atypical meningioma
Meningeal sarcomatosis	9539	Meningeal sarcomatosis

### 2.3 Statistical analysis

Data regarding all cases and incidence rates were obtained from SEER*Stat version 8.3.5 (https://seer.cancer.gov/seerstat/). All incidence rates were age‐adjusted to the 2000 US standard population and expressed per 100 000 person‐years. The Joinpoint Regression Program, version 4.6.0.0 (https://surveillance.cancer.gov/joinpoint/), was used to evaluate the trends in incidence rates during the time accessed, and the simplest joinpoint model allowed by the data was applied. The Monte Carlo permutation method was used to evaluate an apparent change in trend.^8^ A two‐sided *P* value of 0.05 was considered statistically significant.

## 3 RESULTS

Of the 83 030 patients (Table 2) diagnosed with meningioma recorded by SEER‐18 registries during 2004‐2015, women (60 869 [73.3%]) and white people (65 372 [78.7%]) made up the majority. Meningioma patients were mainly older than 40 years old, with 25 670 (30.9%) cases in the 40‐59 years old age bracket, 35 491 (42.7%) cases who were 60‐79 years old, and 16 273 (19.6%) cases who were 80 years old or older. The most common histologic types were meningioma NOS (69 884 [84.2%]), meningothelial meningioma (4628 [5.6%]), and atypical meningioma (2986 [3.6%]). Of all meningioma patients, there were 78 440 (94.5%) cases with WHO grade I, 3568 (4.3%) cases with WHO grade II, and just 1022 (1.2%) cases for WHO grade III. Stratification by tumor size revealed that there were 44 268 (53.3%) cases with tumor size ≤3 cm, 15 123 (18.2%) cases with tumor size >3 to ≤5 cm, and 7789 (9.4%) cases with tumor size >5 cm. However, tumor size was unknown for approximately 19.1% of cases.

**Table 2:** Age‐adjusted incidence of meningioma (2004‐2015): The SEER‐18 registry database

Characteristic	Cases, No. (%)	Rate	95% CI
Overall	83 030 (100)	7.92	7.86‐7.97
Sex			
Male	22 161 (26.7)	4.75	4.68‐4.81
Female	60 869 (73.3)	10.66	10.57‐10.75
Race			
White	65 372 (78.7)	7.82	7.76‐7.88
Black	9825 (11.8)	9.52	9.32‐9.71
AIAN	516 (0.6)	4.76	4.33‐5.23
API	6391 (7.7)	6.56	6.40‐6.73
Age at diagnosis			
<20	370 (0.4)	0.13	0.12‐0.14
20‐39	5226 (6.3)	1.96	1.90‐2.01
40‐59	25 670 (30.9)	8.72	8.61‐8.83
60‐79	35 491 (42.7)	26.20	25.93‐26.48
≥80	16 273 (19.6)	46.83	46.11‐47.55
Histologic type			
Meningioma, NOS	69 884 (84.2)	6.68	6.63‐6.73
Meningothelial	4628 (5.6)	0.44	0.42‐0.45
Fibrous	1519 (1.8)	0.14	0.14‐0.15
Psammomatous	913 (1.1)	0.09	0.08‐0.09
Angiomatous	460 (0.6)	0.04	0.04‐0.05
Hemangioblastic	—	—	—
Transitional	2219 (2.7)	0.21	0.20‐0.22
Clear cell	381 (0.5)	0.04	0.03‐0.04
Atypical	2986 (3.6)	0.28	0.27‐0.29
Meningeal sarcomatosis	28 (‐)	—	—
WHO grade			
I	78 440 (94.5)	7.48	7.43‐7.54
II	3568 (4.3)	0.34	0.33‐0.35
III	1022 (1.2)	0.10	0.09‐0.10
Tumor size, cm			
≤3	44 268 (53.3)	4.23	4.19‐4.27
>3 to ≤5	15 123 (18.2)	1.44	1.42‐1.46
>5	7789 (9.4)	0.74	0.72‐0.75
Unknown	15 850 (19.1)	1.51	1.49‐1.54

Abbreviations: AIAN, American Indian/Alaskan Native; API, Asian/Pacific Islander; SEER, Surveillance, Epidemiology, and End Results.

*Note* Rates were calculated as number of cases per 100 000 person‐years and age‐adjusted to the 2000 US standard population. ‐ Statistic suppressed because of fewer than 16 cases annually.

Age‐adjusted incidence rates according to demographic and tumor characteristics are represented in Table 2. The overall age‐adjusted incidence rate of meningioma was 7.92 (95% CI, 7.86‐7.97) per 100 000 person‐years during 2004‐2015. The incidence rate of women was about two times higher than that of men (10.66 vs 4.75 per 100 000 person‐years). In terms of race, black people (9.52 per 100 000 person‐years) had the highest incidence, followed by white people (7.82 per 100 000 person‐years), Asian/Pacific Islander (APIs) (6.56 per 100 000 person‐years), and American Indian/Alaskan Native (AIANs) (4.76 per 100 000 person‐years). The incidence rate increased sharply with age, from 0.13 per 100 000 person‐years for patients <20 years old to 46.83 per 100 000 person‐years for those ≥80 years old. When stratifying by histologic type, the incidence rate of meningioma NOS was highest at 6.68 per 100 000 person‐years, while the incidence rates of other histological types were extremely low. In terms of WHO grade, grade I meningioma had the highest incidence at 7.48 per 100 000 person‐years and grade III meningioma had the lowest incidence at just 0.10 per 100 000 person‐years. According to tumor size, the age‐adjusted incidence of meningioma was 4.23 per 100 000 person‐years for tumors ≤3 cm, 1.44 per 100 000 person‐years for tumors >3 to ≤5 cm, and 0.74 per 100 000 person‐years for tumors >5 cm.

**Figure 1:**
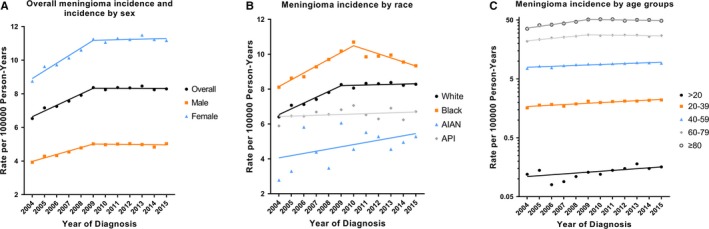
Trends in annual meningioma incidence rates by demographic characteristics (2004‐2015). A, shows overall meningioma incidence rates, and incidence by sex. B, shows meningioma incidence rates by race. C, shows meningioma incidence rates by age groups. All rates are age‐adjusted to the 2000 US standard population. Abbreviations: AIAN, American Indian/Alaskan Native; API, Asian/Pacific Islander

In the period from 2004 to 2015, trends in meningioma incidence according to demographic and tumor characteristics are shown in Table 3 and the joinpoint program divided them into trends 1 to 3. Meningioma incidence rates were increased by an average of 1.9% (95% CI, 1.0‐2.7) per year during the study period (from 6.53 per 100 000 person‐years in 2004 to 8.29 per 100 000 person‐years in 2015). We found the rates increased by 4.6% (95% CI, 3.4‐5.9) annually between 2004 and 2009, but were stable from 2009 to 2015 (Figure 1A). Meningioma incidence rates increased for white people, both sexes and all age groups. The incidence rates in men and women showed similar trends (they increased rapidly between 2004 and 2009, and remained stable between 2009 and 2015) (Figure 1A). There were also no significant changes in incidence rates among patients over 60 years old between 2009 and 2015 (Figure 1C). However, there was a significantly reduced incidence rate for black patients (APC, −2.3% [95% CI, −3.4 to −1.3]) during 2010‐2015 (Figure 1B).

**Table 3:** Trends in meningioma incidence rates (2004‐2015): The SEER‐18 registry database

Characteristic	Overall (2004‐2015)		Trend								
			1			2			3		
	APC (95% CI)	*P*	Year	APC (95% CI)	*P*	Year	APC (95% CI)	*P*	Year	APC (95% CI)	*P*
Overall	1.9 (1.0 to 2.7)	＜.001	2004‐2009	4.6 (3.4 to 5.9)	＜.001	2009‐2015	0 (−0.8 to 0.8)	.969			
Sex											
Male	1.8 (0.9 to 2.7)	.001	2004‐2009	4.7 (3.2 to 6.1)	＜.001	2009‐2015	‐0.2 (−1.1 to 0.8)	.679			
Female	2.0 (1.1 to 2.8)	＜.001	2004‐2009	4.6 (3.2 to 6.1)	＜.001	2009‐2015	0.2 (−0.8 to 1.1)	.688			
Race											
White	2.0 (1.2 to 2.9)	＜.001	2004‐2009	4.6 (3.2 to 6.1)	＜.001	2009‐2015	0.2 (−0.7 to 1.2)	.589			
Black	1.1 (−0.1 to 2.4)	.070	2004‐2010	4.3 (3.3 to 5.2)	＜.001	2010‐2015	‐2.3 (−3.4 to −1.3)	.001			
AIAN^a^	2.7 (−1.1 to 6.7)	.150									
API^a^	0.4 (−0.5 to 1.3)	.402									
Age at diagnosis											
<20^a^	3.6 (0.5 to 6.9)	.029									
20‐39^a^	2.5 (1.8 to 3.3)	＜.001									
40‐59^a^	1.8 (1.3 to 2.4)	＜.001									
60‐79	1.5 (0.5 to 2.5)	.008	2004‐2009	4.8 (3.0 to 6.6)	＜.001	2009‐2015	‐0.7 (−1.8 to 0.5)	.207			
≥80	2.4 (1.0 to 3.8)	.004	2004‐2009	7.0 (4.9 to 9.1)	＜.001	2009‐2015	‐0.6 (−1.9 to 0.7)	.289			
Histologic type											
Meningioma, NOS	2.5 (1.5 to 3.4)	＜.001	2004‐2009	5.5 (4.0 to 7.0)	＜.001	2009‐2015	0.5 (−0.5 to 1.4)	.280			
Meningothelial^a^	−1.3 (−2.5 to −0.1)	.041									
Fibrous	−5.8 (−7.6 to −3.9)	＜.001	2004‐2012	−3.7 (−5.7 to −1.6)	.004	2012‐2015	−15.7 (−25.6 to −4.3)	.015			
Psammomatous^a^	−2.4 (−5.0 to 0.3)	.080									
Angiomatous^a^	−1.4 （−4.2 to 1.4）	.286									
Transitional^a^	−5.0 (−6.9 to −3.0)	＜.001									
Clear cell^a^	3.0 (0.2 to 5.9)	.040									
Atypical^a^	4.0 (3.0 to 5.0)	＜.001									
WHO grade											
I	1.9 (1.0 to 2.8)	＜.001	2004‐2009	4.9 (3.5 to 6.3)	＜.001	2009‐2015	−0.1 (−1.0 to 0.8)	.799			
II	2.8 (1.8 to 3.9)	＜.001	2004‐2008	7.1 (3.8 to 10.6)	.004	2008‐2011	‐2.6 (−11.2 to 6.8)	.471	2011‐2015	5.4 (2.6 to 8.3)	.006
III^a^	−5.6 (−8.5 to −2.6)	.002									
Tumor size, cm											
≤3	4.9 (3.3 to 6.6)	＜.001	2004‐2009	10.6 (8.2 to 13.1)	＜.001	2009‐2015	1.6 (0.2 to 2.9)	.027			
>3 to ≤5	2.5 (1.4 to 3.7)	＜.001	2004‐2009	5.9 (3.0 to 8.9)	.002	2009‐2015	0.3 (−1.5 to 2.2)	.682			
>5^a^	3.2 (1.9 to 4.5)	＜.001									
Unknown^a^	−7.5 (−8.9 to −6.0)	＜.001									

Abbreviations: AIAN, American Indian/Alaskan Native; APC, annual percent change; API, Asian/Pacific Islander; SEER, Surveillance, Epidemiology, and End Results.

*Note* Each time period is determined by Joinpoint Program when a statistically significant change in the APC occurred.

a Denotes only an overall trend for this subgroup after joinpoint analysis.

**Figure 2:**
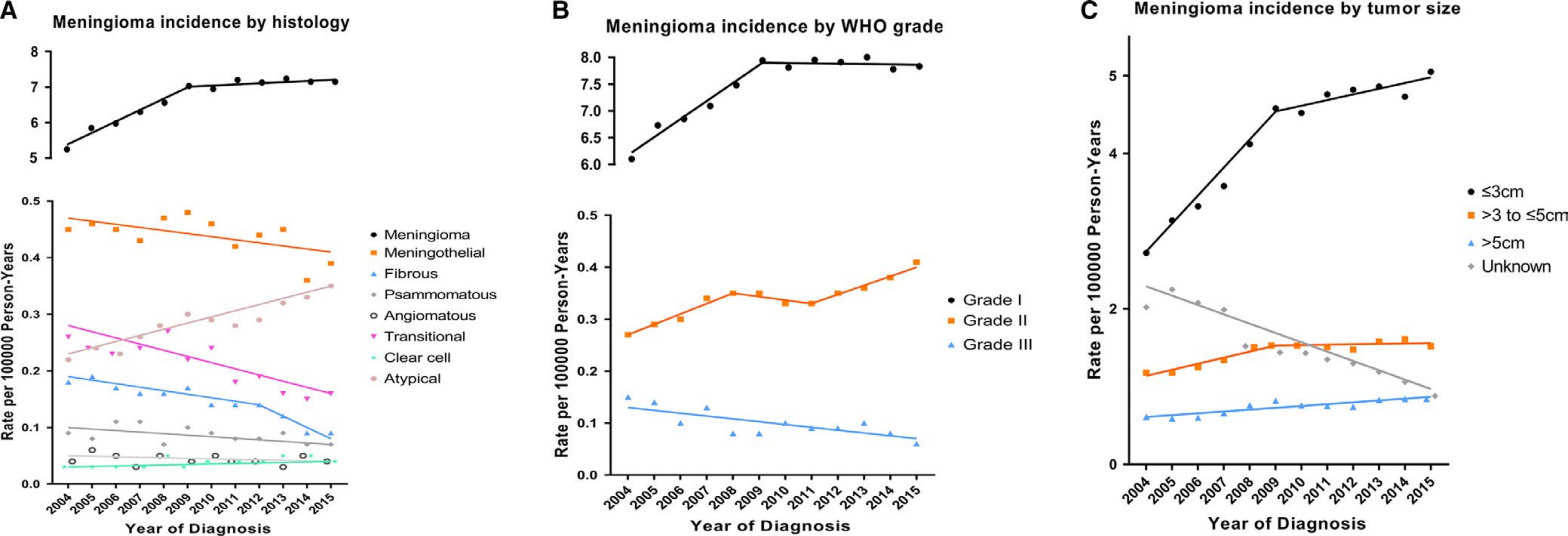
Trends in annual meningioma incidence rates by tumor characteristics (2004‐2015). A, shows meningioma incidence rates by histologic type. B, shows meningioma incidence rates by WHO grade. C, shows meningioma incidence rates by tumor size. All rates are age‐adjusted to the 2000 US standard population. Hemangioblastic meningioma and Meningeal sarcomatosis were not shown due to <16 cases in the time interval

**Figure 3:**
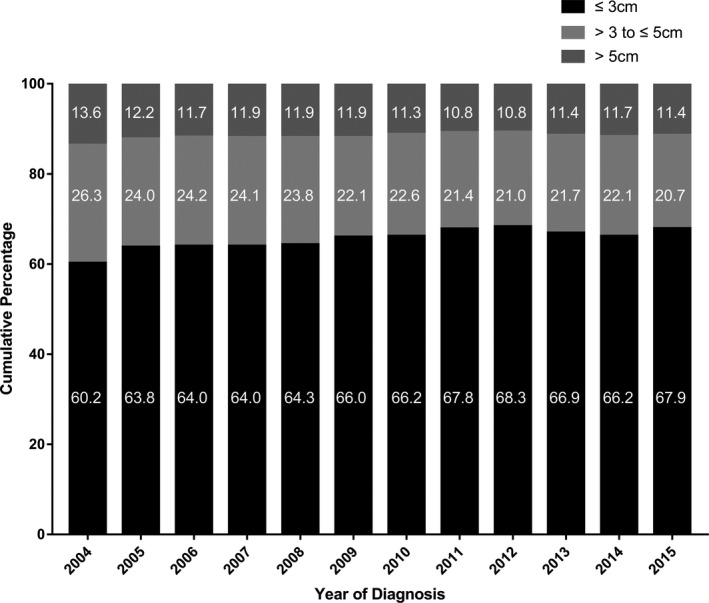
Percentage of known tumor size for meningioma patients by year of diagnosis. Percentages are showed inside the bars

During the study period, incidence rates significantly increased for meningioma NOS (APC, 2.5% [95% CI, 1.5‐3.4]), clear cell meningioma (APC, 3.0% [95% CI, 0.2‐5.9]), and atypical meningioma (APC, 4.0% [95% CI, 3.0‐5.0]), whereas there was a significant decrease in incidence rates for meningothelial meningioma (APC, −1.3% [95% CI, −2.5 to −0.1]), fibrous meningioma (APC, −5.8% [95% CI, −7.6 to −3.9]), and transitional meningioma (APC, −5.0% [95% CI, −6.9 to −3.0]) (Figure 2A). When stratifying by WHO grade, the incidence rates of grade I and grade II meningioma increased by 1.9% (95% CI, 1.0‐2.8) and 2.8% (95% CI, 1.8‐3.9) per year, respectively, but grade III meningioma incidence decreased significantly (APC, −5.6% [95% CI, −8.5 to −2.6]) (Figure 2B). There were significantly increased incidence rates for every tumor size category (Figure 2C). However, the incidence rate of meningioma with unknown tumor size decreased by 7.5% (95% CI, −8.9 to −6.0), which may indicate that the increase in incidence of meningioma with known size was overestimated, and we therefore compared the proportion of cases with known tumor size (Figure 3). Results revealed that the proportion of cases with tumor size less than 3 cm increased, and reached a peak of 68.3% in 2012. The proportion of cases with tumor size between 3 and 5 cm or >5 cm decreased.

**Figure 4:**
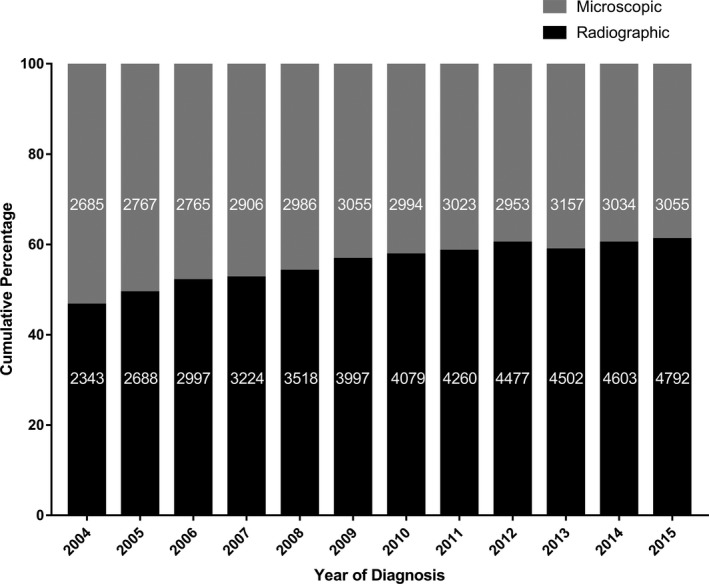
Diagnostic confirmation of meningioma by year of diagnosis. Frequencies are listed inside the bars

Our research also explored the diagnostic confirmation method (Figure 4). The interesting finding was that there were more cases with microscopic diagnosis than those with radiographic diagnosis in the earliest period of our research. However, the proportion of radiographic diagnosis has increased in recent year, even exceeding 60% of the total in 2012, 2014, and 2015.

The annual number of cases and age‐adjusted incidence rates from 2004 to 2015 are shown in Table S1‐S6 in the supplementary data.

## 4 DISCUSSION

Many studies have reported an increase in the incidence of meningioma in recent decades, not only in the United States,^3^ but also in many other countries.^9^, ^10^, ^11^, ^12^, ^13^ However, not all cancer registries in the United States were required to collect data on nonmalignant tumors until 2004, which restricted us to conduct a longitudinal analysis. Thus, we aimed to investigate temporal trends in the incidence of meningioma by demographic and tumor characteristics using updated 12‐year data from SEER registries. Our main finding was that overall meningioma incidence increased significantly from 2004 to 2009 (about 4.9% per year), but remained stable from 2009 to 2015. In addition to overall incidence, we also found that many APCs increased up to 2009 and then remained stable until 2015, for example, both sexes, white people, age ≥60 years old, meningioma NOS, WHO grade I, and medium‐sized (＞3 to ≤5 cm) meningioma. This finding may be related to the changes in population characteristics, the widespread use of diagnostic techniques, changes in tumor classification and risk factors.

There was also a period of significant increase in the incidence rates in our study, consistent with previous studies. To our knowledge, the incidence of meningioma increases sharply with age and peaks at 46.83 per 100 000 person‐years in patients above 80 years old in our study, which was dozens of times higher than young patients. Interestingly, the incidence rate of meningioma in the elderly patients (>60 years old) did not increase between 2009 and 2015, consistent with the overall trend. However, the incidence of meningioma in young people showed a significantly increasing trend during 2004‐2015. We used age‐adjusted incidence, thus eliminating the difference in age distribution among the population. However, an aging population may contribute to an increase in crude incidence rates. A wider availability of CT and MRI examinations was responsible for the increased in incidence of meningioma. The effects of this aspect may continue into this century, and may lead to an increase in the possibility to detect incidental meningioma. A related study reported asymptomatic incidental meningioma was the most frequent brain tumor in the general population, with a prevalence between 0.29% and 0.90%.^14^, ^15^ After the significant increase in detecting meningioma linked to more powerful imaging equipment (such as CT and MRI), the increase in meningioma incidence will reach a plateau level. This may be a plausible explanation for the stable overall incidence between 2009 and 2015.

In addition, SEER registries collected meningioma data starting from 2004 and there may be bias at an early stage. The observed increase in incidence may be partly attributed to improved accuracy and documentation of meningioma in SEER registries partly. Another important change was a minor adjustment regarding meningioma in the WHO central nervous system classification, which upgraded WHO I meningioma with microscopic brain invasion to WHO II in 2007, and downgraded WHO III meningioma with brain invasion but without anaplasia to WHO I or II in 2000.^5^ Although this change would not affect the trend in overall incidence, our results also reflected the fact that the incidence of WHO II meningioma increased again from 2011 to 2015 after remaining stable during 2008‐2011. The decline in the incidence of WHO III meningiomas could be due to changes in WHO classification (2000), as well as improvement in diagnostic accuracy of meningioma, resulting in some malignant meningiomas diagnosed as nonmalignant.

We also found significant predominance in the incidence of meningiomas in female and black patients, consistent with previous reports. Incidence of meningioma in females was more than twice that in males. A number of studies^16^, ^17^, ^18^, ^19^ have suggested sex hormones and genetic differences between males and females were responsible for the differences in meningioma incidence. Another study also reported that meningiomas are related to breast cancer, uterine fibroids, and endometrioses, diseases that are associated with female hormones.^20^ Interestingly, the incidence trends and the magnitude of APCs of males and females were very similar during 2004‐2015 according to our findings. We also identified trends in meningioma incidence by race. Black patients had the highest incidence, and the AIAN population had the lowest incidence, consistent with the report by Achey et al^4^ The reasons for these differences were not only genetic or environmental factors, but also inequalities in health‐care delivery.^21^ Perhaps the concern for the black population has increased in recent years, which may account for the significant decrease in the incidence of meningioma in blacks.

Moreover, some extrinsic risk factors play an important role in the occurrence of meningioma. The only identified risk factor linking to an increase in meningioma is ionizing radiation. Research on ionizing radiation was mainly focused on the tinea capitis radiotherapy studies,^22^, ^23^, ^24^ atomic bomb survivor studies,^25^, ^26^ and medical or occupational exposure.^27^, ^28^, ^29^ Both high doses and low doses of ionizing radiation increase the incidence of meningioma and the latency periods shorten with increasing doses. A large amount of research has been devoted to studying the relationship between cell phones and brain tumor risk.^30^, ^31^ No studies have found that the use of cell phone is associated with a higher risk of meningioma. Other risk factors including the environment, genes and lifestyle have been researched with inconclusive results. High‐quality research is thus essential in the future to integrate these risk factors in order to obtain conclusions.

We must acknowledge several limitations in our study. As this is a retrospective analysis, we can only speculate on the trends in meningioma incidence and the underlying factors. All our data comes from the medical records of the SEER registries, which have documented meningioma data since 2004. Continuous improvement in case records and reports may increase the observed incidence and bias in registries records, which could affect our results. The SEER database only covers 27.8% of the US population, thus not representing the whole US population. Furthermore, only patients diagnosed with meningioma for the first time were enrolled in this study, ignoring recurrent meningioma which could be more malignant. This may lead to an increase in the incidence of WHO I meningioma and a decrease in the incidence of WHO II and III meningioma. In addition, our analysis was limited by the available variables for each patient. Many factors not documented by SEER registries may substantially contribute to the incidence of meningiomas, such as environmental exposures, lifestyle, and meningioma detection methods. Finally, a large proportion of meningiomas is of unknown size, which may lead to an overestimation of the incidence of other size classifications. It is therefore necessary to continue to track trends in meningioma incidence to confirm whether the trends that we observed are sustainable.

## 5 CONCLUSION

In this study, we provide an updated analysis of incidence rates and temporal trends for meningioma by demographic and tumor characteristics in the US population during 2004‐2015. We found the overall incidence increased by 4.6% annually between 2004 and 2009, and remained stable during 2009‐2015. Incidence and trends of meningioma varied significantly by demographic and tumor characteristics, and many subgroups had similar trends to overall incidence. These findings could be related to changes in population characteristics, the widespread use of diagnostic techniques, changes in tumor classification and risk factors, etc, in the US population. Further studies are needed to monitor the incidence of meningioma to determine if the observed incidence trends are persistent.

## CONFLICT OF INTEREST

The authors declare no conflict of interest.
