# Supplementary material for: Trends in intracranial meningioma incidence in the United States, 2004‐2015
Source: Cancer Med. 2019 Sep 1;8(14):6458–67. doi: 10.1002/cam4.2516 (PMC6797634; doi:10.1002/cam4.2516)
Supplement: Supplementary file 1 [file CAM4-8-6458-s001.docx]

| Table S1: Meningioma incidence rates during 2004-2015, overall and according to sex | | | | | | |
| --- | --- | --- | --- | --- | --- | --- |
| Year of diagnosis | Overall | | Sex | | | |
|  |  |  | Male | | Female | |
|  | No. cases | Rate | No. cases | Rate | No. cases | Rate |
| 2004 | 5167 | 6.53 | 1376 | 3.93 | 3791 | 8.75 |
| 2005 | 5588 | 7.16 | 1478 | 4.29 | 4110 | 9.62 |
| 2006 | 5900 | 7.25 | 1556 | 4.33 | 4344 | 9.72 |
| 2007 | 6273 | 7.56 | 1671 | 4.54 | 4602 | 10.13 |
| 2008 | 6680 | 7.91 | 1789 | 4.78 | 4891 | 10.60 |
| 2009 | 7219 | 8.37 | 1917 | 5.02 | 5302 | 11.25 |
| 2010 | 7268 | 8.24 | 1946 | 4.97 | 5322 | 11.06 |
| 2011 | 7509 | 8.37 | 2001 | 5.01 | 5508 | 11.30 |
| 2012 | 7623 | 8.35 | 2057 | 5.03 | 5566 | 11.23 |
| 2013 | 7871 | 8.46 | 2075 | 4.98 | 5796 | 11.49 |
| 2014 | 7866 | 8.24 | 2080 | 4.83 | 5786 | 11.23 |
| 2015 | 8066 | 8.29 | 2215 | 5.03 | 5851 | 11.17 |

Rates were calculated as number of cases per 100,000 person-years and age-adjusted to the 2000 U.S. standard population

| Table S2: Meningioma incidence rates during 2004-2015 according to race | | | | | | | | |
| --- | --- | --- | --- | --- | --- | --- | --- | --- |
| Year of diagnosis | White | | Black | | AIAN | | API | |
|  | No. cases | Rate | No. cases | Rate | No. cases | Rate | No. cases | Rate |
| 2004 | 4130 | 6.41 | 612 | 8.11 | 19 | 2.78 | 379 | 5.89 |
| 2005 | 4484 | 7.07 | 607 | 8.63 | 27 | 3.29 | 432 | 6.46 |
| 2006 | 4686 | 7.12 | 670 | 8.71 | 41 | 5.82 | 443 | 6.45 |
| 2007 | 4949 | 7.41 | 745 | 9.28 | 38 | 4.39 | 481 | 6.69 |
| 2008 | 5287 | 7.81 | 792 | 9.70 | 29 | 3.47 | 498 | 6.55 |
| 2009 | 5700 | 8.26 | 854 | 10.18 | 54 | 6.06 | 536 | 6.82 |
| 2010 | 5664 | 8.06 | 916 | 10.70 | 46 | 4.55 | 570 | 7.06 |
| 2011 | 5920 | 8.33 | 893 | 9.85 | 55 | 5.52 | 557 | 6.53 |
| 2012 | 6002 | 8.31 | 913 | 9.89 | 52 | 5.28 | 561 | 6.29 |
| 2013 | 6130 | 8.38 | 951 | 9.95 | 47 | 4.55 | 642 | 6.91 |
| 2014 | 6145 | 8.22 | 939 | 9.55 | 52 | 4.95 | 611 | 6.24 |
| 2015 | 6275 | 8.28 | 933 | 9.34 | 56 | 5.28 | 681 | 6.71 |

Abbreviations: AIAN: American Indian/Alaskan Native; API: Asian/Pacific Islander

Rates were calculated as number of cases per 100,000 person-years and age-adjusted to the 2000 U.S. standard population

| Table S3: Meningioma incidence rates during 2004-2015 according to age groups | | | | | | | | | | |
| --- | --- | --- | --- | --- | --- | --- | --- | --- | --- | --- |
| Year of diagnosis | ＜20 | | 20-39 | | 40-59 | | 60-79 | | ≥80 | |
|  | No. cases | Rate | No. cases | Rate | No. cases | Rate | No. cases | Rate | No. cases | Rate |
| 2004 | 29 | 0.12 | 364 | 1.61 | 1724 | 7.53 | 2131 | 21.70 | 919 | 35.43 |
| 2005 | 33 | 0.14 | 385 | 1.79 | 1870 | 8.22 | 2245 | 23.30 | 1055 | 40.70 |
| 2006 | 20 | 0.08 | 408 | 1.84 | 1852 | 7.72 | 2493 | 24.81 | 1127 | 41.51 |
| 2007 | 22 | 0.09 | 381 | 1.72 | 2019 | 8.39 | 2641 | 25.55 | 1210 | 43.57 |
| 2008 | 26 | 0.11 | 418 | 1.88 | 2116 | 8.69 | 2801 | 26.49 | 1319 | 46.62 |
| 2009 | 31 | 0.13 | 466 | 2.09 | 2195 | 8.89 | 3060 | 27.95 | 1467 | 50.85 |
| 2010 | 29 | 0.12 | 438 | 1.98 | 2219 | 8.89 | 3070 | 27.17 | 1512 | 51.33 |
| 2011 | 33 | 0.14 | 453 | 2.06 | 2270 | 9.03 | 3217 | 27.65 | 1536 | 51.33 |
| 2012 | 36 | 0.15 | 464 | 2.10 | 2311 | 9.16 | 3327 | 27.74 | 1485 | 48.61 |
| 2013 | 41 | 0.18 | 465 | 2.10 | 2375 | 9.41 | 3445 | 27.68 | 1545 | 50.13 |
| 2014 | 34 | 0.15 | 486 | 2.16 | 2387 | 9.36 | 3395 | 26.09 | 1564 | 49.94 |
| 2015 | 36 | 0.16 | 498 | 2.19 | 2332 | 9.22 | 3666 | 27.10 | 1534 | 48.36 |

Rates were calculated as number of cases per 100,000 person-years and age-adjusted to the 2000 U.S. standard population

| Table S4: Meningioma incidence rates during 2004-2015 according to histology | | | | | | | | | | |
| --- | --- | --- | --- | --- | --- | --- | --- | --- | --- | --- |
| Year of diagnosis | Meningioma, NOS | | Meningothelial | | Fibrous | | Psammomatous | | Angiomatous | |
|  | No. cases | Rate | No. cases | Rate | No. cases | Rate | No. cases | Rate | No. cases | Rate |
| 2004 | 4134 | 5.25 | 362 | 0.45 | 152 | 0.18 | 71 | 0.09 | 34 | 0.04 |
| 2005 | 4551 | 5.85 | 366 | 0.46 | 150 | 0.19 | 64 | 0.08 | 44 | 0.06 |
| 2006 | 4855 | 5.98 | 366 | 0.45 | 140 | 0.17 | 92 | 0.11 | 44 | 0.05 |
| 2007 | 5210 | 6.30 | 367 | 0.43 | 131 | 0.16 | 88 | 0.11 | 29 | 0.03 |
| 2008 | 5523 | 6.56 | 411 | 0.47 | 139 | 0.16 | 59 | 0.07 | 37 | 0.05 |
| 2009 | 6043 | 7.03 | 417 | 0.48 | 152 | 0.17 | 85 | 0.10 | 39 | 0.04 |
| 2010 | 6115 | 6.95 | 406 | 0.46 | 125 | 0.14 | 76 | 0.09 | 44 | 0.05 |
| 2011 | 6438 | 7.20 | 386 | 0.42 | 130 | 0.14 | 79 | 0.08 | 34 | 0.04 |
| 2012 | 6500 | 7.13 | 403 | 0.44 | 127 | 0.14 | 81 | 0.08 | 37 | 0.04 |
| 2013 | 6733 | 7.24 | 422 | 0.45 | 106 | 0.12 | 83 | 0.09 | 31 | 0.03 |
| 2014 | 6827 | 7.15 | 339 | 0.36 | 83 | 0.09 | 66 | 0.07 | 46 | 0.05 |
| 2015 | 6955 | 7.15 | 383 | 0.39 | 84 | 0.09 | 69 | 0.07 | 41 | 0.04 |

Rates were calculated as number of cases per 100,000 person-years and age-adjusted to the 2000 U.S. standard population

| Table S4: Meningioma incidence rates during 2004-2015 according to histology (continued) | | | | | | | | | | |
| --- | --- | --- | --- | --- | --- | --- | --- | --- | --- | --- |
| Year of diagnosis | Hemangioblastic | | Transitional | | Clear cell | | Atypical | | Meningeal sarcomatosis | |
|  | No. cases | Rate | No. cases | Rate | No. cases | Rate | No. cases | Rate | No. cases | Rate |
| 2004 | - | - | 211 | 0.26 | 26 | 0.03 | 171 | 0.22 | - | - |
| 2005 | - | - | 197 | 0.24 | 22 | 0.03 | 186 | 0.24 | - | - |
| 2006 | - | - | 191 | 0.23 | 27 | 0.03 | 183 | 0.23 | - | - |
| 2007 | - | - | 201 | 0.24 | 25 | 0.03 | 219 | 0.26 | - | - |
| 2008 | - | - | 231 | 0.27 | 38 | 0.05 | 237 | 0.28 | - | - |
| 2009 | - | - | 196 | 0.22 | 26 | 0.03 | 259 | 0.30 | - | - |
| 2010 | - | - | 210 | 0.24 | 32 | 0.04 | 258 | 0.29 | - | - |
| 2011 | - | - | 161 | 0.18 | 32 | 0.04 | 247 | 0.28 | - | - |
| 2012 | - | - | 172 | 0.19 | 32 | 0.04 | 269 | 0.29 | - | - |
| 2013 | - | - | 152 | 0.16 | 44 | 0.05 | 295 | 0.32 | - | - |
| 2014 | - | - | 145 | 0.15 | 40 | 0.05 | 318 | 0.33 | - | - |
| 2015 | - | - | 152 | 0.16 | 37 | 0.04 | 344 | 0.35 | - | - |

Rates were calculated as number of cases per 100,000 person-years and age-adjusted to the 2000 U.S. standard population

- Statistic suppressed due to <16 cases annually

| Table S5: Meningioma incidence rates during 2004-2015 according to WHO grade | | | | | | |
| --- | --- | --- | --- | --- | --- | --- |
| Year of diagnosis | Grade I | | Grade II | | Grade III | |
|  | No. cases | Rate | No. cases | Rate | No. cases | Rate |
| 2004 | 4829 | 6.10 | 218 | 0.27 | 120 | 0.15 |
| 2005 | 5257 | 6.73 | 224 | 0.29 | 107 | 0.14 |
| 2006 | 5579 | 6.85 | 244 | 0.30 | 77 | 0.10 |
| 2007 | 5888 | 7.09 | 282 | 0.34 | 103 | 0.13 |
| 2008 | 6317 | 7.48 | 294 | 0.35 | 69 | 0.08 |
| 2009 | 6848 | 7.94 | 304 | 0.35 | 67 | 0.08 |
| 2010 | 6882 | 7.81 | 298 | 0.33 | 88 | 0.10 |
| 2011 | 7135 | 7.95 | 293 | 0.33 | 81 | 0.09 |
| 2012 | 7225 | 7.91 | 316 | 0.35 | 82 | 0.09 |
| 2013 | 7446 | 8.00 | 334 | 0.36 | 91 | 0.10 |
| 2014 | 7426 | 7.78 | 360 | 0.38 | 80 | 0.08 |
| 2015 | 7608 | 7.83 | 401 | 0.41 | 57 | 0.06 |

Rates were calculated as number of cases per 100,000 person-years and age-adjusted to the 2000 U.S. standard population

| Table S6: Meningioma incidence rates during 2004-2015 according to tumor size | | | | | | | | |
| --- | --- | --- | --- | --- | --- | --- | --- | --- |
| Year of diagnosis | ≤3cm | | ＞3 to ≤5cm | | ＞5cm | | Unknown | |
|  | No. cases | Rate | No. cases | Rate | No. cases | Rate | No. cases | Rate |
| 2004 | 2146 | 2.72 | 934 | 1.18 | 483 | 0.61 | 1604 | 2.02 |
| 2005 | 2445 | 3.14 | 919 | 1.18 | 466 | 0.59 | 1758 | 2.25 |
| 2006 | 2689 | 3.32 | 1018 | 1.25 | 493 | 0.60 | 1700 | 2.08 |
| 2007 | 2957 | 3.58 | 1112 | 1.34 | 550 | 0.66 | 1654 | 1.99 |
| 2008 | 3469 | 4.12 | 1282 | 1.51 | 642 | 0.76 | 1287 | 1.52 |
| 2009 | 3939 | 4.58 | 1318 | 1.53 | 711 | 0.82 | 1251 | 1.44 |
| 2010 | 3972 | 4.52 | 1354 | 1.53 | 676 | 0.76 | 1266 | 1.43 |
| 2011 | 4276 | 4.76 | 1348 | 1.51 | 679 | 0.75 | 1206 | 1.35 |
| 2012 | 4399 | 4.82 | 1352 | 1.48 | 694 | 0.74 | 1178 | 1.30 |
| 2013 | 4527 | 4.86 | 1470 | 1.58 | 773 | 0.83 | 1101 | 1.19 |
| 2014 | 4547 | 4.73 | 1518 | 1.61 | 802 | 0.84 | 999 | 1.06 |
| 2015 | 4902 | 5.05 | 1498 | 1.52 | 820 | 0.84 | 846 | 0.88 |

Rates were calculated as number of cases per 100,000 person-years and age-adjusted to the 2000 U.S. standard population
